# The relationships between perfectionism, pathological worry and generalised anxiety disorder

**DOI:** 10.1186/1471-244X-14-98

**Published:** 2014-04-02

**Authors:** Alicia K Handley, Sarah J Egan, Robert T Kane, Clare S Rees

**Affiliations:** 1 School of Psychology and Speech Pathology, Curtin University, GPO Box U1987, Perth, Western Australia 6845, Australia

**Keywords:** Perfectionism, Pathological worry, Generalised anxiety disorder

## Abstract

**Background:**

The relationships between perfectionism, pathological worry and generalised anxiety disorder (GAD) were investigated in a clinical sample presenting for treatment of perfectionism.

**Method:**

This study explored the utility of perfectionism in predicting pathological worry in a sample of individuals with elevated perfectionism and GAD (*n = 36*). Following this, the study examined whether perfectionism could predict a principal GAD diagnosis in the full sample (*n = 42*).

**Results:**

Scores on the perfectionism dimensions Concern over Mistakes, Personal Standards, and Clinical Perfectionism significantly predicted pathological worry among participants with GAD after controlling for gender and depression. The perfectionism dimension Doubts about Actions significantly predicted whether individuals from the full sample received a principal diagnosis of GAD.

**Conclusions:**

These findings support certain dimensions of perfectionism having significant associations with pathological worry and GAD.

## Background

Comprehensive reviews have reported dimensions of perfectionism playing a key role in mood disorders, eating disorders and various anxiety disorders [1]. Perfectionism is significantly elevated in social phobia, obsessive-compulsive disorder (OCD), and panic disorder with agoraphobia compared to controls [1]. Dimensions of perfectionism have also been shown to significantly relate to symptomatology in social phobia [2], OCD [3,4], panic disorder with agoraphobia [5], and post-traumatic stress disorder [6].

To date however, one anxiety disorder that has not received attention in the perfectionism literature is Generalised Anxiety Disorder (GAD). No research has examined the relationship between perfectionism and pathological worry in a clinical sample of individuals with GAD. Pathological worry refers to worry that is perceived to be unremitting, hard to control, excessive and of a distressing nature [7]. It is a defining characteristic of GAD [8]. While studies have investigated the role of perfectionism in pathological worry [7,9-11], these studies did not use clinical samples. Investigating the relationship between perfectionism and pathological worry in a clinical GAD sample is important, as evidence of a significant relationship would provide additional support for perfectionism being a construct that cuts across disorders, namely, a transdiagnostic process [1].

Additionally, no studies have examined whether perfectionism is associated with a principal diagnosis of GAD in a clinical sample with a range of disorders. Evidence of perfectionism being significantly associated with a principal GAD diagnosis and pathological worry would support the utility of future research examining whether treatment of perfectionism can decrease GAD symptomatology [1,12].

Perfectionism has been defined predominantly as a multidimensional construct [13,14]. A 35-item Multidimensional Perfectionism Scale was developed that measures six elements of perfectionism: Concern over Mistakes (CM; concern over errors in performance), Personal Standards (PS; setting high personal goals), Parental Criticism (PC; belief that one’s parents were critical), Parental Expectations (PE; parents holding high expectations for one’s behaviour), Doubts about Actions (DA; doubting oneself and one’s actions), and Organisation (O; neatness and organisation) [13]. An additional 45-item Multidimensional Perfectionism Scale was constructed that measures three elements of perfectionism; Self-Oriented Perfectionism (SOP; setting high goals and standards), Socially-Prescribed Perfectionism (SPP; belief that others expect perfection from oneself), and Other-Oriented Perfectionism (OOP; belief that others should be perfect) [14].

Studies using non-clinical samples have found significant relationships between various components of perfectionism (CM, DA, PE, PC, SPP, and SOP) and pathological worry [7,9-11]. One study using a sample of university students [7] found that a subscale comprising of the maladaptive perfectionism dimensions Concern over Mistakes and Doubts about Actions was significantly correlated with scores on the Penn State Worry Questionnaire (PSWQ) [15] after controlling for anxiety and depression [7]. A second study [11] found that a subscale of the same perfectionism dimensions remained significantly correlated with PSWQ after controlling for experiential avoidance, depression and social anxiety. Such studies support perfectionism having a unique relationship with worry in non-clinical samples [7,11].

The relationship between *maladaptive evaluative concerns* perfectionism (MEC = CM + DA + PE + PC), and anxiety factors has also been examined in students [10]. The anxiety factors consisted of obsessive-compulsive symptoms, social/trait/worry anxiety, and post-traumatic stress. While the correlations between MEC perfectionism and all anxiety factors were significant, the strongest correlation existed between MEC and social/trait/worry anxiety. MEC perfectionism was the only significant predictor of social/trait/worry anxiety after controlling for depression. The authors argued that these findings supported the relationship between perfectionism and social/trait/worry anxiety being important in its own right and not just arising from perfectionism being related to depression [10,16].

Self-Oriented Perfectionism and Socially-Prescribed Perfectionism have been found to have significant positive correlations with the autonomic arousal and worry dimensions of state anxiety in a student sample [17]. An additional study found in a student sample that Self-Oriented Perfectionism and Socially-Prescribed Perfectionism had significant positive correlations with worry on the PSWQ after controlling for gender. Self-Oriented Perfectionism remained a significant predictor of worry after controlling for demographics and intolerance of uncertainty [9]. These studies support Socially-Prescribed Perfectionism and Self-Oriented Perfectionism having significant associations with worry [9,17].

Clinical Perfectionism as measured by the Clinical Perfectionism Questionnaire (CPQ) [18] was found to be a significant positive predictor of anxiety and stress in a student sample after controlling for negative affect, Self-Oriented Perfectionism, Socially-Prescribed Perfectionism, and Other-Oriented Perfectionism [19]. The construct of Clinical Perfectionism has been put forward as an alternative definition of perfectionism to the multidimensional view, and focuses on self-worth being based on the attainment of personal standards [20]. While this study highlighted a key role for Clinical Perfectionism in predicting anxiety and stress in a non-clinical sample [19], the role of Clinical Perfectionism in predicting pathological worry has not been investigated. This would be useful to establish given emerging evidence of the validity of the CPQ as a measure of Clinical Perfectionism [21]. It would help to further investigate the validity of the CPQ, as it has been proposed to be a clinically relevant measure for use in understanding change in the cognitive-behavioural treatment of perfectionism, which has been found to effectively reduce perfectionism and a range of disorders, including eating disorders and depression [22-25].

This study had two aims. The first aim was to examine the relationships between perfectionism dimensions [13,18] and pathological worry in a sample of participants with elevated perfectionism and GAD (*n* = 36) who were participating in a perfectionism treatment trial. Specifically, this study examined whether Concern over Mistakes, Doubts about Actions, and Personal Standards are related to pathological worry as assessed by the PSWQ [15]. This study also investigated the relationship between Clinical Perfectionism and pathological worry as measured by the PSWQ. As Concern over Mistakes and Doubts about Actions most frequently represent the clinical aspects of perfectionism [1], it was predicted that these perfectionism dimensions would each explain a unique proportion of the variability in pathological worry after controlling for gender and depression. As the perfectionism dimension of Personal Standards has been argued to be adaptive [26] and has not predicted symptomatology in non-clinical or most other anxiety disorder samples [1,7,11], it was predicted that Personal Standards would not explain a significant proportion of the variability in pathological worry after controlling for gender and depression. Based on Clinical Perfectionism scores predicting anxiety in a non-clinical sample [19], it was hypothesised that Clinical Perfectionism scores would significantly predict pathological worry and that this would remain significant after controlling for gender and depression.

The second aim was to examine whether perfectionism can significantly predict a principal diagnosis of GAD from a larger sample of individuals (*n = 42*) with elevated perfectionism and a range of diagnoses who presented for perfectionism treatment. As pathological worry is a defining characteristic of GAD [8], it was predicted that the perfectionism dimensions hypothesised to be involved in pathological worry (Concern over Mistakes, Doubts about Actions, Clinical Perfectionism) would significantly predict a principal GAD diagnosis, whereas Personal Standards perfectionism would not.

## Method

### Participants

The first aim was examined using a sample of 36 adults (81% female; 19% male) with elevated perfectionism and GAD. This subset of participants with GAD was from a larger sample (*n = 42*) of individuals with elevated perfectionism and a range of disorders who were at baseline assessment for participation in a perfectionism treatment trial. Participants had self-referred to this treatment trial in response to letters and advertisement fliers distributed to psychologists, psychiatrists, general practitioners, workplaces, and universities throughout the metropolitan area of Perth, Australia. Due to the inclusion criterion of the perfectionism treatment research, all participants had elevated perfectionism as defined by a score of greater than 24.7 on the Concern over Mistakes subscale [13]. Elevated Concern over Mistakes is one of the most clinically relevant aspects of perfectionism, with this scale being related to numerous disorders [1]. The Concern over Mistakes cut-off score was derived by averaging the mean Concern over Mistakes scores from six studies that investigated perfectionism in other anxiety disorder samples as cited in a recent review [1]. Eighty-three per cent of participants had a principal diagnosis of GAD based on rating their worries and anxiety as causing the greatest distress. Seventeen per cent had GAD as their second or third diagnosis. The mean number of disorders per participant was 2.36 (*SD* = 1.10). The mean age was 30.86 years (*SD* = 11.30). The mean level of depression on the BDI-II was 19.44 (*SD =* 11.51), which indicated that the sample on average had a mild level of depression, based on the clinical cut-off ranges of the BDI-II [27].

The second aim was examined using a sample of 42 adults (81% females; 19% males) presenting for perfectionism treatment. All participants had elevated perfectionism as defined by a score of greater than 24.7 on the Concern over Mistakes subscale [13]. Ninety per cent had a current DSM-IV-TR [8] diagnosis of a psychological disorder; and the remaining ten per cent had depression in remission. Seventy-one per cent had a principal diagnosis of GAD. The mean number of disorders per participant was 2.071 (*SD =* 1.26). The mean age was 31.47 years (*SD* = 11.01). The mean level of depression on the BDI-II was 20.29 (*SD =* 12.04), which indicated that the sample on average had a mild level of depression, based on the clinical cut-off ranges of the BDI-II [27].

The perfectionism treatment trial from which the current data is derived has received approval by the Curtin University Human Research Ethics Committee, and is in compliance with the Helsinki Declaration [28]. All participants provided written informed consent to participate in this perfectionism research.

### Measures

#### Frost multidimensional perfectionism scale (FMPS)

The Concern over Mistakes, Personal Standards and Doubts about Actions subscales of the FMPS [13] were used, which have high internal consistency [13], and construct validity [3]. For the sample of 36 participants, Cronbach’s alpha was .91 (CM), .82 (PS), and .73 (DA). For the sample of 42 participants, Cronbach’s alpha was .90 (CM); .81 (PS); and .72 (DA).

#### Clinical Perfectionism Questionnaire (CPQ)

The CPQ [18] assesses one’s level of clinical perfectionism in the past month. Items are rated on 4-point Likert-type scales, and Items 2 and 8 are reverse coded. Higher scores denote higher clinical perfectionism. The CPQ has been reported to have adequate internal consistency, test-retest reliability and validity [20,22,29]. For the sample of 36 participants, Cronbach’s alpha was .74. For the sample of 42 participants, Cronbach’s alpha was .77.

#### Mini international neuropsychiatric interview, version 5.0 (MINI)

The MINI [30] is a structured interview that identifies DSM-IV-TR disorders [8] and was used to determine whether participants met the criteria for a GAD diagnosis. A single interviewer (AKH), who at the time was blind to participants’ scores on the self-report inventories, conducted all structured interviews. AKH has a Master of Clinical Psychology degree and four years of experience administering this measure. While it was not possible to obtain a measure of inter-rater reliability, AKH discussed the diagnoses with the second author (SJE), a clinical psychologist with many years of experience administering this measure, who provided confirmation. The MINI has been shown to have high test-retest reliability, internal consistency, and validity [31].

#### Penn State Worry Questionnaire (PSWQ)

The PSWQ [15] assesses the excessiveness, uncontrollability and generality of clinical worry. Higher scores denote greater clinical worry. The PSWQ has excellent test-retest reliability, internal consistency and validity [15]. For the sample of 36 participants, Cronbach’s alpha was .88. For the sample of 42 participants, Cronbach’s alpha was .91.

#### Beck Depression Inventory-II

The BDI-II [27] measures symptoms of depression and has high internal consistency, test-retest reliability and validity [27]. For the sample of 36 participants Cronbach’s alpha was .92. For the sample of 42 participants, Cronbach’s alpha was .92.

### Statistical methods

Analysis for the first aim involved calculating the means, standard deviations and zero-order correlations for the measures of perfectionism, pathological worry and depression. To determine the degree to which components of FMPS perfectionism [13] predicted worry on the PSWQ [15], a hierarchical multiple linear regression analysis was conducted. Only perfectionism variables that had significant zero-order correlations with PSWQ were entered as predictors.

To determine the degree to which Clinical Perfectionism measured by the CPQ [18] predicted pathological worry on the PSWQ [15], a second hierarchical multiple linear regression analysis was conducted. The relationship between Clinical Perfectionism and PSWQ was explored in a separate linear regression model because the CPQ is a more recent measure and the relationship between Clinical Perfectionism and PSWQ has never been examined.

If gender and depression were found to have significant zero-order correlations with PSWQ, both of the regression models above were to control for gender and depression. Controlling for depression on the final step of each regression model enabled examination of whether any relationships between perfectionism and PSWQ that emerged on previous steps of the model were unique and not due to overlap from a relationship between perfectionism and depression [16].

Analysis for the second aim involved calculating the means and standard deviations for the measures of perfectionism, pathological worry and depression for the full sample and the sample with a principal diagnosis of GAD. The zero-order correlations between gender, the measures of perfectionism, pathological worry, depression and a principal diagnosis of GAD were then calculated. If two or more variables were significantly correlated with a principal diagnosis of GAD, a binary logistic regression analysis was to be conducted [32]. Only the variables that had significant correlations with a principal diagnosis of GAD would be included in the regression model. If gender and depression were correlated with a principal diagnosis of GAD, they would be controlled in the regression model, otherwise they would not be included.

### Control of type I and type II errors

An alpha level of .05 was applied throughout, therefore for each regression model, the probability of a Type I error was 5 per cent. The probability of a Type II error was ascertained by the power of the statistical test [32]. In this study, the most complex regression model contained four predictors. Based on the current sample size, at an alpha level of .05, the four-predictor regression model had an 80 per cent likelihood of capturing ‘moderate to large’ associations between each of the four predictors and the dependent variable [33]. Thus, the probability of failing to capture ‘moderate to large’ associations in the population was 20 per cent [32,33].

## Results

To examine the first aim of the study, Table 1 reports the means, standard deviations and zero-order correlations for gender, the measures of perfectionism, pathological worry and depression. The perfectionism dimensions of CM, PS, and CPQ each had significant moderate correlations with PSWQ. The perfectionism dimension of DA was not significantly correlated with PSWQ (*p* = .07). As gender and depression were each significantly correlated with PSWQ, the regression model controlled for gender and depression. Gender, CM and PS were entered at Step 1 and BDI-II was entered at Step 2. The tolerance values for each predictor in this regression analysis were sufficiently high to suggest that predictors were not multicollinear [32]. As only 34 participants from this sample completed the BDI-II, the regression analysis was conducted with 34 participants.

**Table 1:** Means (standard deviations) and zero-order correlations in participants with a diagnosis of GAD (n = 36)

	**Means (SDs)**	**Gender**	**CM**	**PS**	**DA**	**CPQ**	**PSWQ**	**BDI-II**
Gender	-	-	.30	.12	.00	-.01	.39*	.07
CM	33.19 (6.47)		-	.32	.44**	.52**	.68**	.49**
PS	28.61 (4.46)			-	.51**	.58**	.49**	.38*
DA	15.86 (2.85)				-	.60**	.30	.43*
CPQ	32.19 (8.54)					-	.49**	.56**
PSWQ	67.28 (8.54)						-	.35*
BDI-II^a^	19.44 (11.51)							-

*Note.* CM: Concern over Mistakes subscale [13]; PS: Personal Standards subscale [13]; DA: Doubts about Actions subscale [13]; CPQ: Clinical Perfectionism Questionnaire [18]; PSWQ: Penn State Worry Questionnaire [16]; BDI-II: Beck Depression Inventory-II [27].

^a^Descriptive statistics and correlations for the BDI-II were calculated for 34 participants.

*p < .05; **p < .01.

As seen in Table 2, at Step 1, gender explained a non-significant 5% of the variance in pathological worry (*sr*^*2*^ × 100 = 5, *p =* .057). CM explained a significant 23% of the variance in pathological worry (*sr*^*2*^ × 100 = 23, *p* = .000), where higher CM scores predicted higher pathological worry. PS explained a significant 8% of the variance in pathological worry (*sr*^*2*^ × 100 = 8, *p* = .019) where higher PS predicted higher pathological worry. After adding BDI-II at Step 2, CM explained a significant 20% of the variance in pathological worry (*sr*^*2*^ × 100 = 20, *p* = .000), and PS explained a significant 8% of the variance in pathological worry (*sr*^*2*^ × 100 = 8, *p* = .021). Higher CM and PS scores both predicted higher pathological worry.

**Table 2:** Hierarchical multiple regression analysis predicting pathological worry from perfectionism in a GAD sample (n = 34)

**Outcome (DV)**	**Predictors (IVs)**	***B***	**95% CI**	***β***	***sr***^**2**^	***p*-value**
PSWQ	**Step 1**					
	Gender	4.77	−0.15, 9.70	0.24	.05	.057
	CM	0.66	0.34, 0.98	0.53	.23	.000**
	PS	0.54	0.09, 0.98	0.29	.08	.019*
	*Adjusted R*^2^ = .58, *p* = .000**					
	**Step 2**					
	Gender	4.66	−0.39, 9.70	0.23	.05	.069
	CM	0.69	0.33, 1.05	0.55	.20	.000**
	PS	0.56	0.09, 1.03	0.31	.08	.021*
	BDI-II	−0.04	−0.24, 0.17	−0.05	.00	.706
	▲ *R*^2^ = .002, *p* = .706					
	*Adjusted R*^2^ = .56, *p* = .000**					

*Note. sr*^2^: part-correlation squared; CM: Concern over Mistakes subscale [13]; PS: Personal Standards subscale [13]; DA: Doubts about Actions subscale [13]; PSWQ: Penn State Worry Questionnaire [16]; BDI-II: Beck Depression Inventory-II [27].

*p < .05; **p < .01.

For the second hierarchical linear regression analysis, gender and CPQ score were entered at Step 1, and BDI-II was entered at Step 2. The tolerance values for each predictor in this regression analysis were adequately high to suggest that predictors were not multicollinear [32]. As only 34 participants from this sample completed the BDI-II, the regression analysis was conducted with 34 participants.

As seen in Table 3, at Step 1, gender explained a significant 20% of the variance in pathological worry (*sr*^*2*^ × 100 = 20, *p* = .003), where females reported higher pathological worry than males. CPQ uniquely explained a significant 18% of the variance in pathological worry (*sr*^*2*^ × 100 = 18, *p* = .005). Higher CPQ scores indicated higher pathological worry. After adding BDI-II at Step 2, CPQ explained a significant 9% of the variance in pathological worry (*sr*^*2*^ × 100 = 9, *p* = .040), where higher CPQ scores indicated higher pathological worry.

**Table 3:** Hierarchical multiple regression analysis predicting pathological worry from CPQ scores in a GAD sample (n = 34)

**Outcome (DV)**	**Predictors (IVs)**	***B***	** 95% CI**	***β***	***sr***^**2**^	***p*-value**
PSWQ	**Step 1**					
	Gender	9.00	3.24, 14.76	0.45	.20	.003**
	CPQ	0.68	0.23, 1.14	0.42	.18	.005**
	*Adjusted R*^2^ = .35, *p* = .000**					
	**Step 2**					
	Gender	8.84	3.00, 14.69	0.44	.19	.004**
	CPQ	0.58	0.03, 1.13	0.37	.09	.040*
	BDI-II	0.08	−0.17, 0.33	0.12	.01	.506
	▲ *R*^2^ = .01, *p* = .506					
	*Adjusted R*^2^ = .34, *p* = .001**					

*Note. sr*^2^: part-correlation squared; CPQ: Clinical Perfectionism Questionnaire [18]; PSWQ: Penn State Worry Questionnaire [16]; BDI-II: Beck Depression Inventory-II [27].

*p < .05; **p < .01.

To examine the second aim of the study, Table 4 reports the means and standard deviations for the measures of perfectionism, pathological worry and depression for the full sample and the sample with a principal diagnosis of GAD. Table 5 reports the zero-order correlations between gender, the measures of perfectionism, pathological worry, depression and a principal diagnosis of GAD. The perfectionism dimension of DA had a significant moderate correlation with a principal diagnosis of GAD. The perfectionism dimensions CM, PS and CPQ did not have significant correlations with a principal diagnosis of GAD. As the principal diagnosis of GAD was not significantly correlated with gender or depression, the binary logistic regression model would not provide additional information to that of the zero-order correlations, thus it was not conducted.

**Table 4:** Means (standard deviations) for the full sample (n=42) and the sample with a principal GAD diagnosis (n=30)

	**PSWQ**	**CM**	**PS**	**DA**	**CPQ**	**BDI-II**^**a**^
Full sample	65.77 (9.68)	33.09 (5.99)	28.53 (4.27)	15.63 (2.79)	31.47 (5.60)	18.51 (11.23)
Sample with principal GAD	67.50 (8.57)	33.47 (6.07)	29.07 (4.23)	16.43 (2.56)	32.23 (5.35)	20.29 (12.04)

*Note.* PSWQ: Penn State Worry Questionnaire [16]; CM: Concern over Mistakes subscale [13]; PS: Personal Standards subscale [13]; DA: Doubts about Actions subscale [13]; CPQ: Clinical Perfectionism Questionnaire [18]; BDI-II: Beck Depression Inventory-II [27].

^a^Descriptive statistics for the BDI-II were calculated for 38 participants in the full sample and 28 participants in the principal GAD diagnosis sample.

**Table 5:** Zero-order correlations between gender, perfectionism, pathological worry, depression, and a principal GAD diagnosis (n = 42)

	**Gender**	**CM**	**PS**	**DA**	**CPQ**	**PSWQ**	**BDI-II**	**Principal GAD**
Gender	-	.27	.13	.00	.01	.34*	-.03	.10
CM		-	.31*	.42**	.48**	.54**	.49**	.10
PS			-	.45**	.55**	.43**	.35*	.25
DA				-	.60**	.42**	.46**	.46**
CPQ					-	.54**	.55**	.19
PSWQ						-	.37*	.26
BDI-II^a^							-	.14
Principal GAD								-

*Note.* CM: Concern over Mistakes subscale [13]; PS: Personal Standards subscale [13]; DA: Doubts about Actions subscale [13]; CPQ: Clinical Perfectionism Questionnaire [18]; PSWQ: Penn State Worry Questionnaire [16]; BDI-II: Beck Depression Inventory-II [27].

^a^Correlations for the BDI-II were calculated for 38 participants as 4 participants did not complete the measure.

*p < .05; **p < .01.

## Discussion

In reference to the first aim of the study, perfectionism as measured by Concern over Mistakes, Personal Standards and CPQ were each significant positive predictors of pathological worry after controlling for gender and depression. The significant relationship between Concern over Mistakes and pathological worry in this sample of participants with elevated perfectionism and GAD is consistent with the findings of previous research using non-clinical samples [7,10,11]. The finding of Clinical Perfectionism being a significant predictor of pathological worry adds to the literature by highlighting that a significant association exists between Clinical Perfectionism as measured by the CPQ and pathological worry. Both Concern over Mistakes and Clinical Perfectionism were significantly related to pathological worry after depression was controlled, which provides support for such relationships being real and not just due to the relationship between perfectionism and depression [7,10,11,16].

The significant relationship between Personal Standards and pathological worry that remained after controlling for depression was not expected, and may reflect Personal Standards having different relationships with pathology in clinical and non-clinical samples [7,10,11]. Even so, the current finding is important as it is the first finding of Personal Standards being significantly associated with pathological worry in a clinical sample. It is only the third study to find that Personal Standards is significantly associated with anxiety pathology in a clinical sample, as most previous research has found Personal Standards not to be related to anxiety disorders [1]. Demonstrating a significant relationship between Personal Standards and anxiety symptomatology in individuals with elevated perfectionism and GAD suggests that Personal Standards is not a purely positive aspect of perfectionism as has been argued in previous research [26]. Consistent with this, other research utilising clinical samples has shown Personal Standards to have significant relationships with eating disorder and depressive symptomatology (see [1]).

The current findings of Concern over Mistakes, Personal Standards and Clinical Perfectionism playing a role in pathological worry support perfectionism being a transdiagnostic process [1]. It provides a rationale for future research to examine whether interventions targeting perfectionism can reduce pathological worry in samples of individuals with elevated perfectionism and GAD.

The current study did not find a significant correlation between Doubts about Actions and pathological worry. This is inconsistent with previous research using non-clinical samples [7,10,11]. This may be because previous research only looked at the relationship between the composite variable Concern over Mistakes + Doubts about Actions (CM + DA) in predicting pathological worry. It is therefore possible that the significant relationship between CM + DA and pathological worry in the non-clinical samples may be an artefact of the significant relationship between just Concern over Mistakes and pathological worry. If so, this would be consistent with the present findings of Concern over Mistakes predicting worry on the PSWQ. Another possibility is that due to all participants having elevated perfectionism, the data was influenced by a restriction of range on the study variables, which may have attenuated the relationships between the dependent variables and the predictors [32]. Additionally, the small sample size may have prevented the relationship between Doubts about Actions and pathological worry from reaching statistical significance. As the correlation between Doubts about Actions and pathological worry had a *p* value of .07, it is possible that a Type II error occurred, leading to the exclusion of Doubts about Actions from the first regression model [32]. This requires exploration in future studies.

In reference to the second aim of the study, Doubts about Actions was a significant positive predictor of a principal GAD diagnosis whereas Concern over Mistakes, Personal Standards and Clinical Perfectionism did not significantly predict a principal GAD diagnosis. The predictive utility of Doubts about Actions supports it playing a role in GAD. This is consistent with perfectionism being a transdiagnostic process [1]. Even so, the exact role of Doubts about Actions in a principal GAD diagnosis requires clarification as it did not significantly predict pathological worry, which is a primary symptom of GAD [8]. Given that Doubts about Actions plays a significant role in OCD [3,4], it is possible that the current finding of Doubts about Actions predicting a principal GAD diagnosis may reflect a common cognitive process shared between OCD and GAD. Future research needs to investigate the role of Doubts about Actions in predicting a GAD diagnosis.

It is intriguing that Concern over Mistakes, Personal Standards and Clinical Perfectionism were not significant predictors of a principal GAD diagnosis given the significant predictive utility of these variables in pathological worry. This could again be due to restriction of range on the study variables. Alternatively, since 71% of the sample had a principal GAD diagnosis, the sample may not have been diverse enough for these variables to emerge as significant predictors [32]. Future research needs to be conducted with more diverse samples.

This study contributes to the literature by highlighting that significant relationships exist between specific dimensions of perfectionism, pathological worry and a principal GAD diagnosis in a clinical sample. This finding has clinical relevance as it highlights the need for mental health professionals to include questions about perfectionism when conducting assessments for individuals presenting with GAD symptomatology. The clinician could then include perfectionism in a client’s formulation if it appears to be maintaining the client’s symptoms. These findings provide a rationale for future research to examine whether treatments that target perfectionism can decrease GAD symptomatology in addition to the symptoms of other psychological disorders [1,12]. Nevertheless, limitations of this study warrant discussion. One limitation was that all participants had elevated levels of perfectionism. This may have introduced the bias of restriction of range [32]. Furthermore, findings of Concern over Mistakes, Personal Standards and Clinical Perfectionism predicting pathological worry can only be generalised to individuals with elevated perfectionism and GAD; whereas findings of Doubts about Actions predicting a principal GAD diagnosis can only be generalised to individuals with elevated perfectionism. An additional limitation was the small sample sizes used in this study, which may have resulted in Type II errors [32]. Future research should utilise a larger sample that has a greater range of perfectionism [32]. Additionally, the current study did not utilise a non-clinical control group, thus future research needs to compare the level of perfectionism in a clinical GAD sample to that of healthy controls.

## Conclusion

In sum, the current study found that significant associations exist between certain dimensions of perfectionism, pathological worry and GAD. Such findings have clinical relevance for the assessment of individuals with GAD, and provide impetus for future research to explore whether treatment of perfectionism can ameliorate GAD symptomatology [1,12]. This may hold significant promise for improving treatment outcome in individuals with GAD.

## Abbreviations

GAD: Generalised anxiety disorder; OCD: Obsessive-compulsive disorder; CM: Concern over mistakes subscale; PS: Personal standards subscale; PC: Parental criticism subscale; PE: Parental expectation subscale; DA: Doubts about actions subscale; O: Organisation subscale; SOP: Self-oriented perfectionism; SPP: Socially-prescribed perfectionism; OOP: Other-oriented perfectionism; CM + DA: The combined subscales of concern over mistakes and doubts about actions; PSWQ: Penn State Worry Questionnaire; MEC: Maladaptive evaluative concerns; CPQ: Clinical Perfectionism Questionnaire; FMPS: Frost Multidimensional Perfectionism Scale; MINI: Mini international neuropsychiatric interview; DSM-IV-TR: Diagnostic and statistical manual of psychiatric disorders-IV, text revision; BDI-II: Beck Depression Inventory-II; SD: Standard deviation.

## Competing interests

All authors state that they have no competing interests.

## Authors’ contributions

AKH collected the data, analysed the results, and drafted the manuscript. SJE confirmed the client diagnoses assigned by AKH and helped to revise the manuscript. RTK assisted with statistical analyses and helped to revise the manuscript. CSR helped to revise the manuscript. All authors read and approved the final manuscript.

## Pre-publication history

The pre-publication history for this paper can be accessed here:

http://www.biomedcentral.com/1471-244X/14/98/prepub
